# Supplementary material for: Dual inhibition of AKT‐mTOR and AR signaling by targeting HDAC3 in PTEN‐ or SPOP‐mutated prostate cancer
Source: EMBO Mol Med. 2018 Mar 9;10(4):e8478. doi: 10.15252/emmm.201708478 (PMC5887910; doi:10.15252/emmm.201708478)
Supplement: Supplementary file 1 — Appendix [file EMMM-10-e8478-s001.docx]

**APPENDIX**

**Table of Content**

Table of content………………………………………………………………………………….1

Appendix Table S1………………………………………………………………………………2

Appendix Table S2………………………………………………………………………………3

**Appendix Table S1. DNA sequences for primers**

| Gene | Sequence | Company |
| --- | --- | --- |
| HDAC3 P1 F | CCCGAATTCATGGCCAAGACCGTGGCGTATTTC | IDT |
| HDAC3 P1 R | CCCCTCGAGAAGTCCTGGAAACACTGGGCAGTC | IDT |
| HDAC3 P2 F | CCCGAATTCTTCGAGTTCTGCTCCCGTTACACAG | IDT |
| HDAC3 P2 R | CCCCTCGAGTCCAACTTCATACATGTCACCTGTTC | IDT |
| HDAC3 P3 F | CCCGAATTCGCAGAGAGTGGCCGCTACTATTGTC | IDT |
| HDAC3 P3 R | CCCCTCGAGTTCATATGTCCAACACCGGGCAAC | IDT |
| HDAC3 P4 F | CCCGAATTCACATCTCTGCTGGTAGAAGAGGCC | IDT |
| HDAC3 P4 R | CCCCTCGAGAATCTCCACATCACTTTCCTTGTCG | IDT |
|  |  |  |
| APPL P1 F | CCCGAATTCATGCCGGGGATCGACAAGCTGCCC | IDT |
| APPL P1 R | CCCCTCGAGTGTCTGTTGCATGGTCTCTATATC | IDT |
| APPL P2 F | CCCGAATTCATAGAGGATTTGGAAGTAGCCAGTG | IDT |
| APPL P2 R | CCCCTCGAGGTCTGGGGCAACAAGAGAATCTAG | IDT |
| APPL P3 F | CCCCTCGAGGTCTGGGGCAACAAGAGAATCTAG | IDT |
| APPL P3 R | CCCCTCGAGTTTTTGTTTTTCTGATGCCCTACG | IDT |
| APPL P4 F | CCCGAATTCGAAATAGAGAGAGTAAAAGAGAAGC | IDT |
| APPL P4 R | CCCCTCGAGTTATGCTTCTGATTCTCTCTTCTTTC | IDT |

**Appendix Table S2. DNA sequences for siRNAs**

| Gene | Sequence | Cat. No |
| --- | --- | --- |
| siRNA HDAC3 SMARTpool |  | M-003496-00-0020 |
| siRNA HDAC3-01 | GGAAAGCGAUGUGGAGAUU | D-003496-01 |
| siRNA HDAC3-02 | AAAGCGAUGUGGAGAUUUA | D-003496-02 |
| siRNA HDAC3-03 | GCAUUGAUGACCAGAGUUA | D-003496-03 |
| siRNA HDAC3-04 | GGAAUGCGUUGAAUAUGUC | D-003496-04 |
|  |  |  |
| siRNA human APPL1 SMARTpool |  | M-005138-01-0005 |
| siRNA APPL1-1 | GAGUGGAUCUGUACAAUAA | D-005138-01 |
| siRNA APPL1-2 | CAACACACCUGACCUCAAA | D-005138-02 |
| siRNA APPL1-3 | GAACAAAGUCGGUUGAUAG | D-005138-03 |
| siRNA APPL1-3 | CCACAGCUAUUUCCAACUA | D-005138-04 |
|  |  |  |
| siRNA PTEN SMARTpool |  | M-003023-02-0005 |
| si-PTEN-05 | GUGAAGAUCUUGACCAAUG | D-003023-05 |
| si-PTEN-06 | GAUCAGCAUACACAAAUUA | D-003023-06 |
| si-PTEN-07 | GGCGCUAUGUGUAUUAUUA | D-003023-07 |
| si-PTEN-07 | GUAUAGAGCGUGCAGAUAA | D-003023-08 |
