# Supplementary material for: Dual inhibition of AKT‐mTOR and AR signaling by targeting HDAC3 in PTEN‐ or SPOP‐mutated prostate cancer
Source: EMBO Mol Med. 2018 Mar 9;10(4):e8478. doi: 10.15252/emmm.201708478 (PMC5887910; doi:10.15252/emmm.201708478)
Supplement: Supplementary file 2 — Expanded View Figures PDF [file EMMM-10-e8478-s002.pdf]

Expanded View Figures

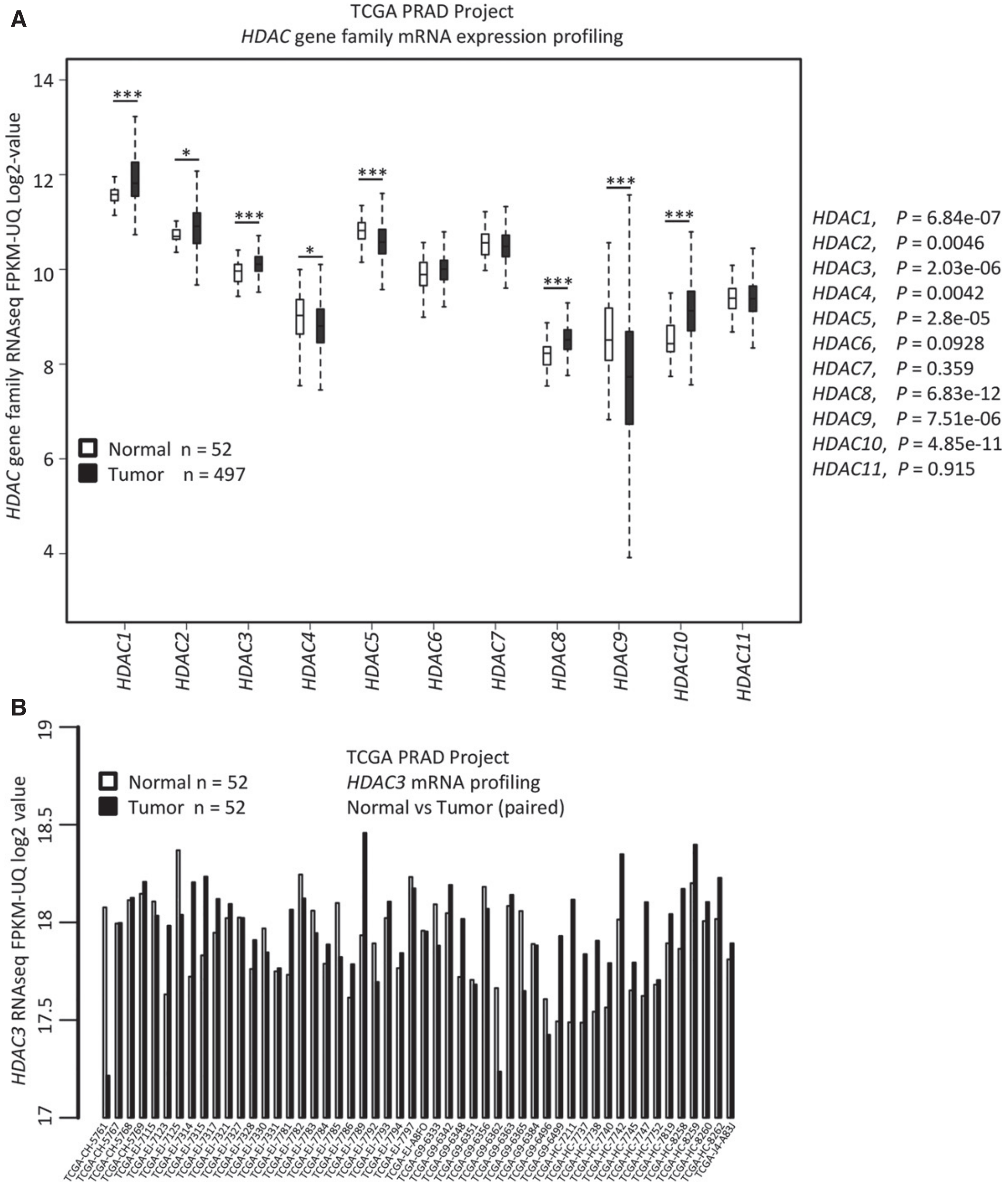

Figure EV1. HDAC3 is overexpressed in prostate cancer patient specimens.

A The mRNA level of 11 HDAC gene family members was compared between normal and tumor tissues (the mRNA expression data were extracted from the TCGA project). *P*-values were performed between normal tissues ($n = 52$) with tumor tissues ($n = 497$) for each gene by Wilcoxon rank sum test with continuity correction and shown alongside the graph. The description for box plot is the same as the figure legend in Fig 7H.

B The mRNA level of *HDAC3* gene was compared between paired normal and cancer tissues for individual patient. Normal/tumor paired samples were available only in 52 patients in the TCGA cohort.

© 2018 The Authors

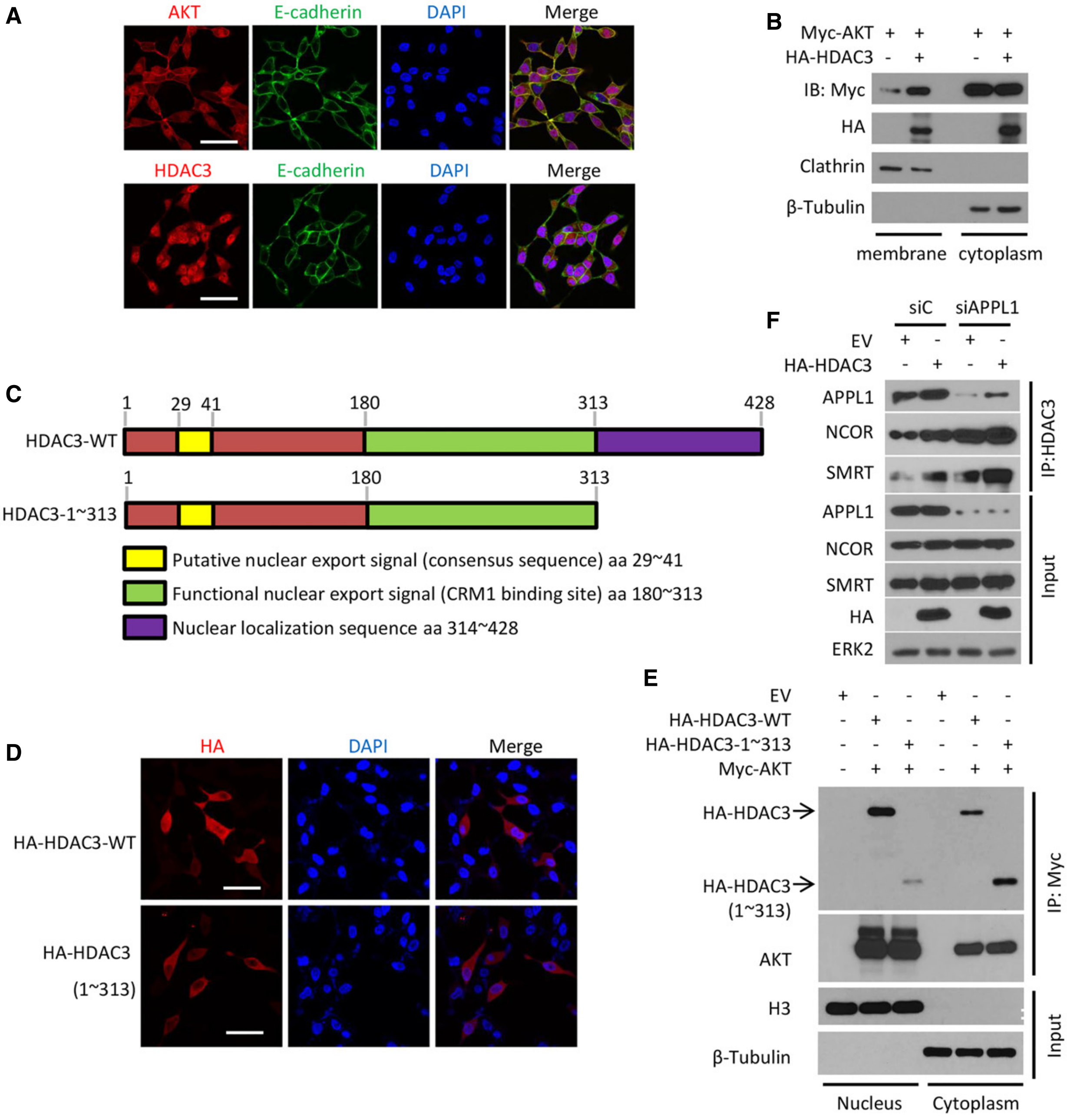

Figure EV2. The C-terminal region (aa 1–313) in HDAC3 is indispensable for its cytoplasm exportation that is a prerequisite for AKT phosphorylation and AR upregulation.

A LNCaP cells were treated with 10 ng of IGF-1 for 30 m and harvested for immunofluorescent cytochemistry (IFC) with P-AKT-S473, E-cadherin, and HDAC3. Cell nuclei were counterstained with DAPI in IFC. Scale bars, 50 μm.

B C4-2 cells were transfected with the indicated plasmids, and then, cytoplasm membrane and nuclear proteins were isolated followed by IP and Western blots with the indicated antibodies.

C An illustration depicts functional domains of HDAC3 including the regions for nuclear export and localization sequences (modified from the following website: http://atlasgeneticsoncology.org/Genes/GC_HDAC3.html).

D, E LNCaP cells were transfected with HA-tagged wild-type HDAC3 or truncated mutant (aa 1–313) for 24 h followed by IFC with anti-HA antibody (D) or Western blots with indicated antibodies (E). Scale bars for images in (D), 50 μm.

F C4-2 cells were transfected with a pool of siRNA of *APLL1* and the indicated plasmids. The cells were harvested for IP and Western blots with the indicated antibodies.

Source data are available online for this figure.

© 2018 The Authors

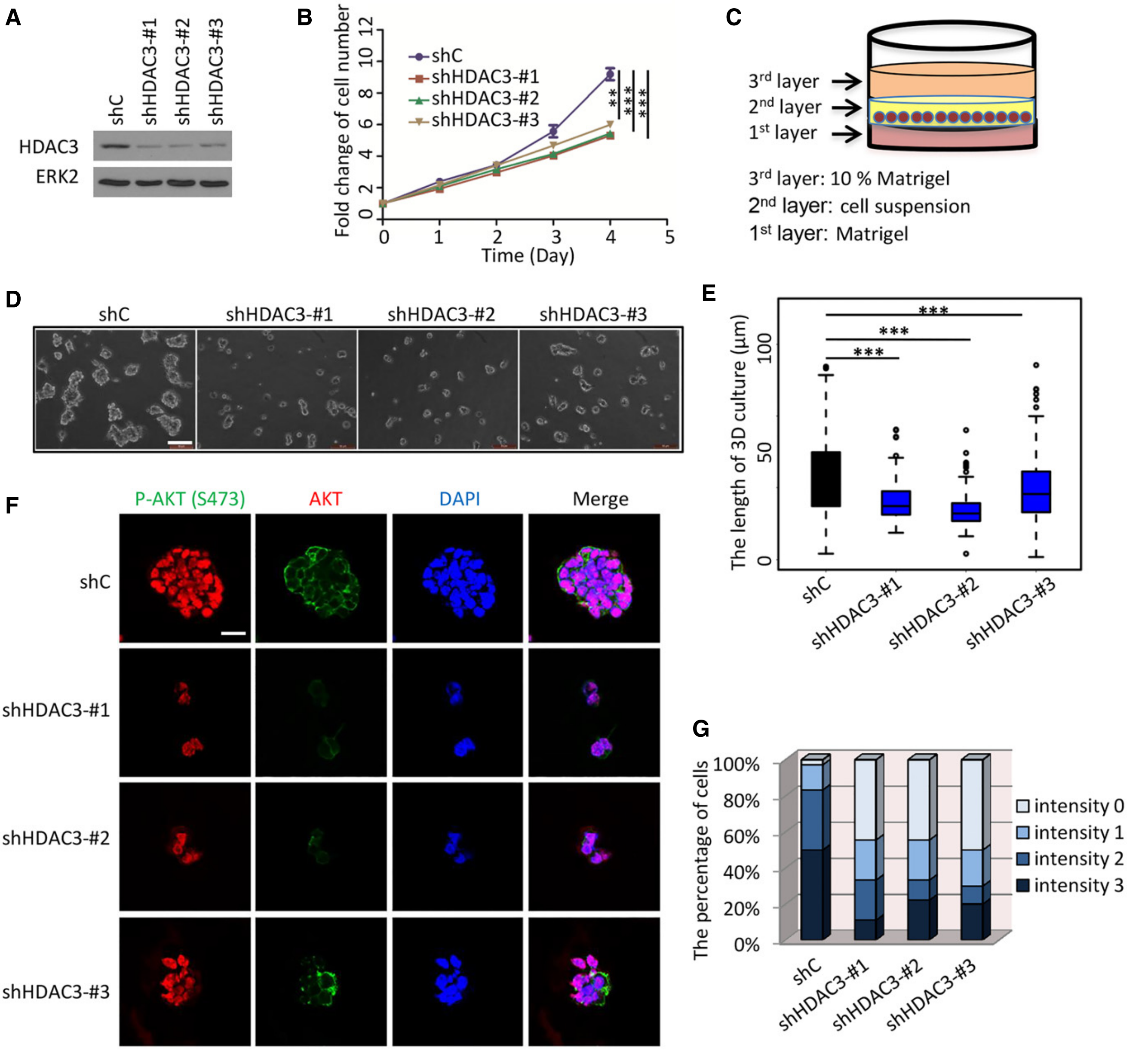

Figure EV3. ***HDAC3* knockdown undermines AKT phosphorylation and prostate cancer cell growth in 3D culture.**

A C4-2 cells stably infected with lentivirus for control or *HDAC3*-specific shRNAs were harvested for Western blots with the indicated antibodies.

B C4-2 cells were infected with lentivirus as in (A) and subjected to cell growth analysis using a 2D cell culture system. The cell growth was measured in triplicate for each of three independent experiments. Data are shown as means ± SEM. shC versus shHDAC3-#1: ****P* = 6.0e-04, shC versus shHDAC3-#2: ****P* = 6.5e-04, shC versus shHDAC3-#3: ***P* = 0.0012 comparing the cell growth at day 4 by the unpaired two-tailed Student's *t*-test.

C An illustration shows the workflow of a 3D Matrigel culture system.

D, E C4-2 cells were infected with lentivirus as in (A) and subjected to cell growth analysis using a 3D cell culture system. The representative images from 3D cultures at day 5 are shown in (D) with quantitative data in a box plot (E). Scale bar, 100 μm. In graph (E), the description for box plot is the same as the figure legend in Fig 7H. Data are shown as means ± SEM. shC (*n* = 321) versus shHDAC3-#1 (*n* = 397): ****P* = 2.02e-31, shC (*n* = 321) versus shHDAC3-#2 (*n* = 402): ****P* = 4.43e-54, shC (*n* = 321) versus shHDAC3-#3 (*n* = 334): ****P* = 4.35e-06 were performed by Wilcoxon rank sum test with continuity correction.

F, G C4-2 cells in 3D cultures at day 5 were subjected for IFC analysis for expression of total and S473 phosphorylated AKT. The representative images of AKT phosphorylation (S473) are shown in (F) with quantitative data in (G). Scale bar for images in (F), 20 μm.

Source data are available online for this figure.

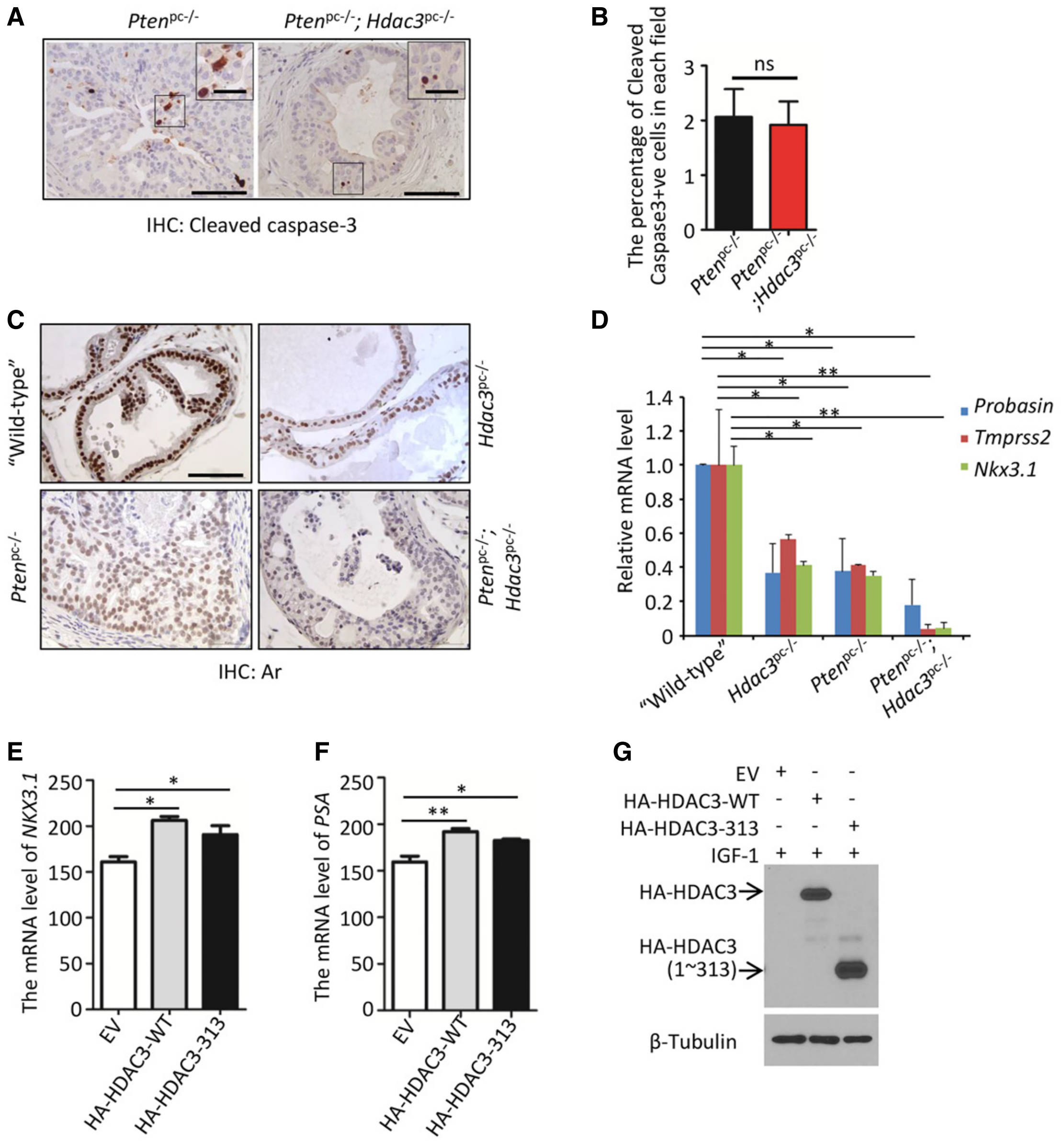

Figure EV4. Loss of Hdac3 impairs Ar signaling in mouse prostate tissues and has no effect on apoptosis.

A, B Representatives of cleaved caspase-3 staining in prostate tissues from *Pten*$^{pc-/-}$ and *Pten*$^{pc-/-}$;*Hdac3*$^{pc-/-}$ mice at age of 4 months are shown in (A) with the quantitative data in (B). Scale bar: 50 μm; scale bar in the inset: 10 μm. The "ns" stands for "no significant difference". Data are shown as means ± SEM. *Pten*$^{pc-/-}$ (n = 6) versus *Pten*$^{pc-/-}$;*Hdac3*$^{pc-/-}$ (n = 6), P = 0.8339 was performed by the unpaired two-tailed Student's t-test.

C Ar protein expression was examined by IHC in prostate tissues of "wild-type", *Hdac3*$^{pc-/-}$, *Pten*$^{pc-/-}$ and *Pten*$^{pc-/-}$;*Hdac3*$^{pc-/-}$ mice at age of 4 months. Scale bar, 50 μm.

D RT–qPCR analysis of mRNA levels of Ar target genes (*Probasin*, *Tmprss2*, and *Nkx3.1*). Data shown as means ± SEM (n = 6 mice for each group). For *Probasin*, "wild-type" versus *Hdac3*$^{pc-/-}$, *P = 0.034; "wild-type" versus *Pten*$^{pc-/-}$, *P = 0.043; and "wild-type" versus *Pten*$^{pc-/-}$;*Hdac3*$^{pc-/-}$, *P = 0.016. For *Tmprss2*, "wild-type" versus *Hdac3*$^{pc-/-}$, *P = 0.018; "wild-type" versus *Pten*$^{pc-/-}$, *P = 0.022; and "wild-type" versus *Pten*$^{pc-/-}$;*Hdac3*$^{pc-/-}$, **P = 0.0027. For *Nkx3.1*, "wild-type" versus *Hdac3*$^{pc-/-}$, *P = 0.018; "wild-type" versus *Pten*$^{pc-/-}$, *P = 0.015; and "wild-type" versus *Pten*$^{pc-/-}$;*Hdac3*$^{pc-/-}$, **P = 0.007. Statistical analysis was performed by the unpaired two-tailed Student's t-test.

E–G LNCaP cells were transfected the indicated plasmids and treated with 10 ng/ml of IGF-1 for 0.5 h before harvesting. The mRNA expression of AR targets (*NKX3.1* and *PSA*) normalized to *GAPDH* is shown in (E, F), and expression of transfected plasmids was examined by Western blots (G). The RT–qPCR was performed in triplicate for each sample. Data are shown as means ± SEM. For *NKX3.1*, EV versus HA-HDAC3-WT, *P = 0.013; EV versus HA-HDAC3-313, *P = 0.014. For *PSA*, EV versus HA-HDAC3-WT, **P = 0.009; EV versus HA-HDAC3-313, *P = 0.02. Statistical analysis was performed by the unpaired two-tailed Student's t-test.

Source data are available online for this figure.

 © 2018 The Authors

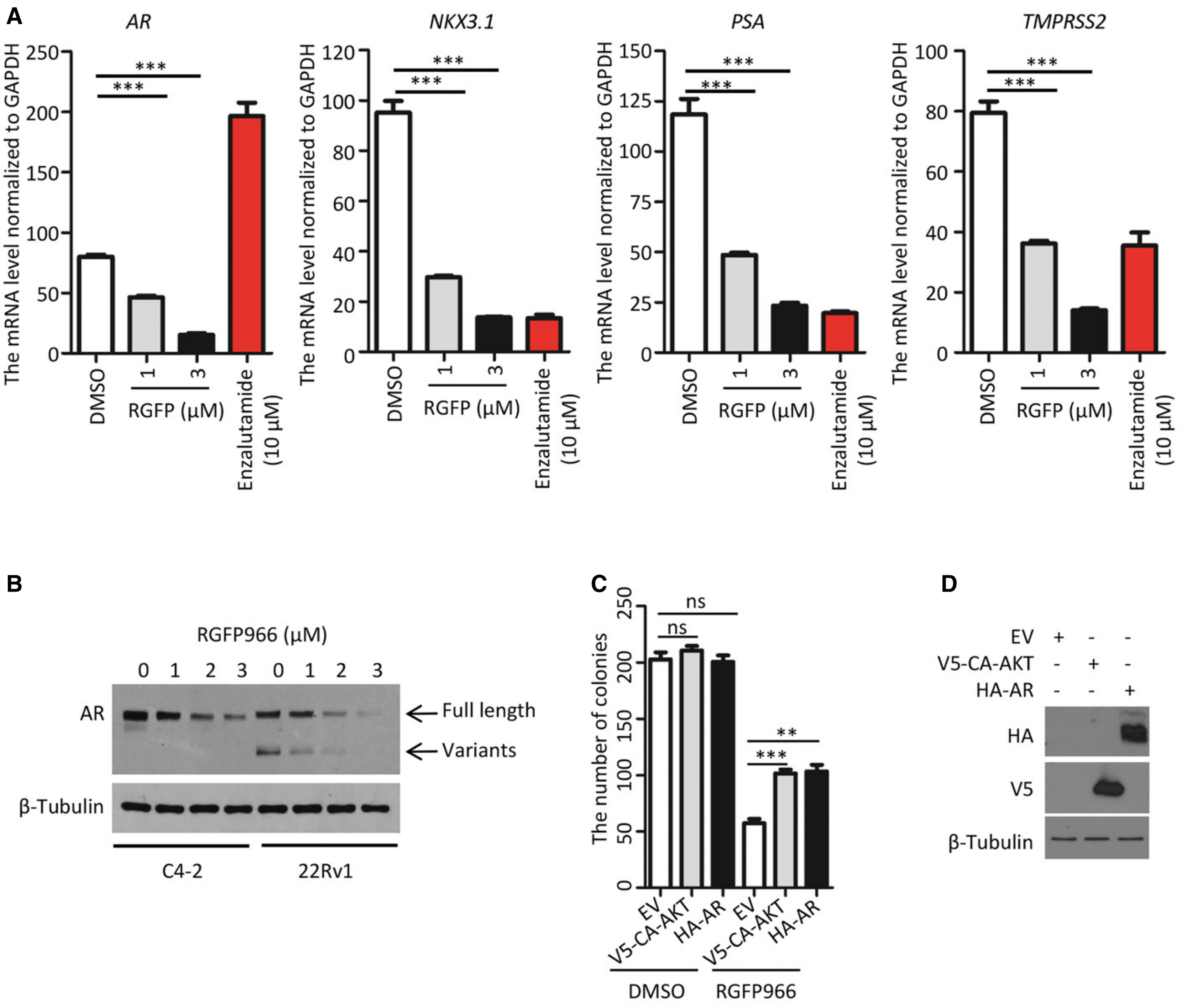

Figure EV5. The sensitivity to HDAC3 inhibitor is dependent on the status of AKT and AR.

A C4-2 cells were treated with vehicle (DMSO); different concentrations of HDAC3 inhibitor RGFP966 or the antiandrogen enzalutamide (positive control) for 24 h and cells were harvested for RT–qPCR analysis of mRNA levels of *AR* and its downstream target genes (*NKX3.1*, *PSA*, and *TMPRSS2*). The RT–qPCR was performed in triplicate for each sample. Data represents means ± SEM. For *AR* mRNA level, DMSO versus RGFP966 (1 μM): ***P = 9.42e-05, DMSO versus RGFP966 (3 μM): ***P = 6.88e-06; for NKX3.1 mRNA level, DMSO versus RGFP966 (1 μM): ***P = 1.47e-04, DMSO versus RGFP966 (3 μM): ***P = 6.12e-05; for *PSA* mRNA level, DMSO versus RGFP966 (1 μM): ***P = 8.24e-04, DMSO versus RGFP966 (3 μM): ***P = 2.54e-04; for *TMPRSS2* mRNA level, DMSO versus RGFP966 (1 μM): ***P = 3.60e-04, DMSO versus RGFP966 (3 μM): ***P = 6.81e-05 were performed using the unpaired two-tailed Student's *t*-test.

B C4-2 and 22Rv1 cells were treated with different concentrations of the HDAC3 inhibitor RGFP966 for 24 h and harvested for Western blot analysis with the indicated antibodies.

C, D C4-2 cells transfected with V5-CA-AKT or HA-AR were treated with two times of IC50 (2.5 μM) of RGFP966. The number of colonies after 10-day treatment is shown in (C), and transfected proteins were analyzed by Western blots (D). The RT–qPCR was performed in triplicate for each sample. Data represent means ± SEM. For RGFP treatment, EV versus V5-CA-AKT, ***P = 0.0009; EV versus HA-AR, **P = 0.0026. Statistical analysis was performed using the unpaired two-tailed Student's *t*-test.

Source data are available online for this figure.

© 2018 The Authors
