# Supplementary figures and images for: Dual inhibition of AKT‐mTOR and AR signaling by targeting HDAC3 in PTEN‐ or SPOP‐mutated prostate cancer
Source: EMBO Mol Med. 2018 Mar 9;10(4):e8478. doi: 10.15252/emmm.201708478 (PMC5887910; doi:10.15252/emmm.201708478)

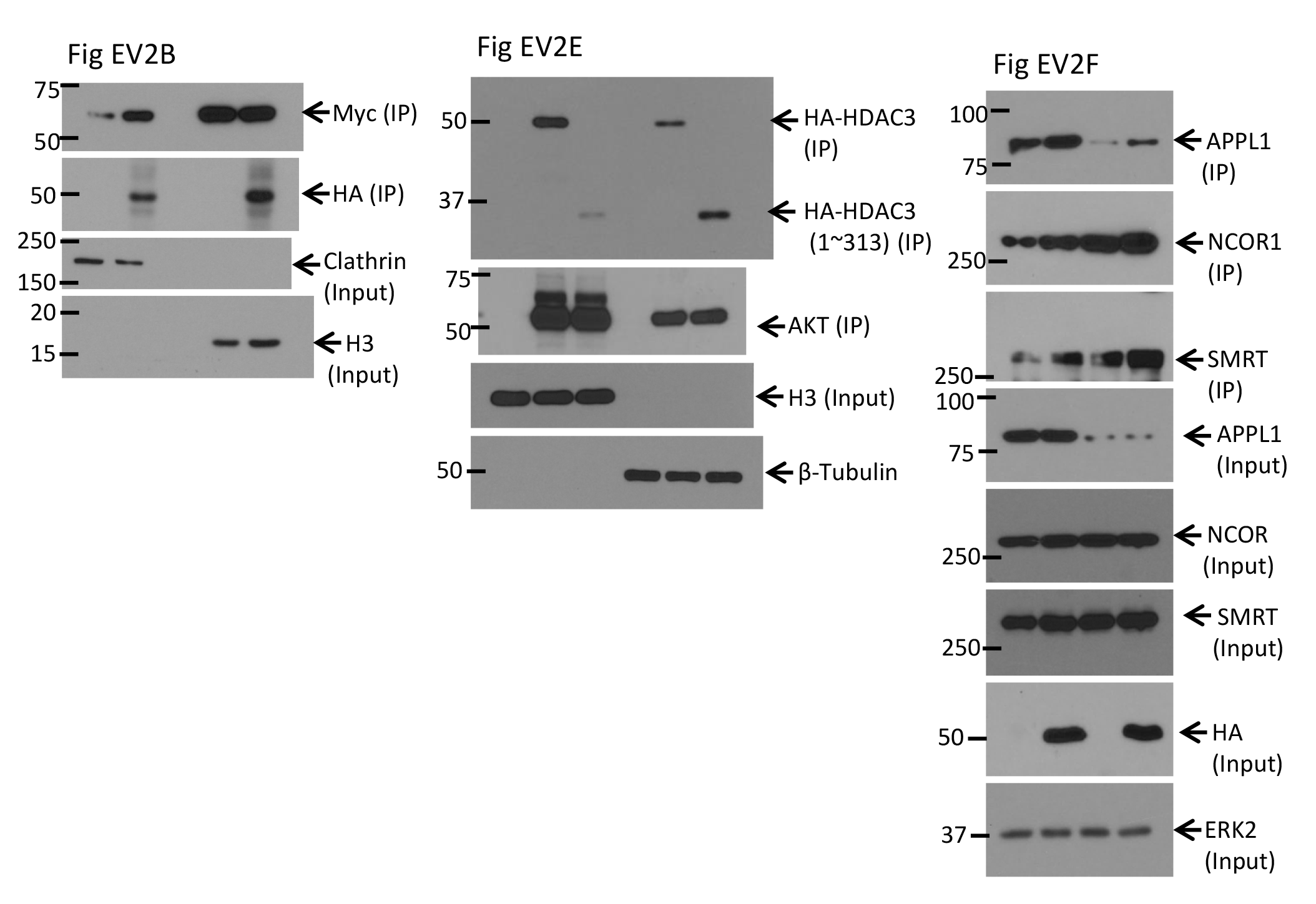

Supplement: Supplementary file 3 — Source Data for Expanded View [file EMMM-10-e8478-s011.zip › Source_data_EV2.tif]

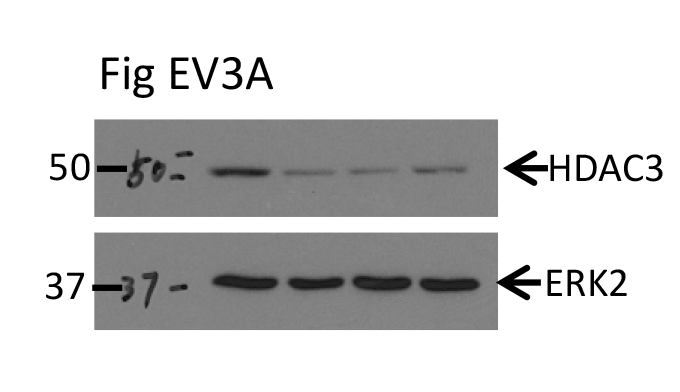

Supplement: Supplementary file 3 — Source Data for Expanded View [file EMMM-10-e8478-s011.zip › Source_data_EV3.tif]

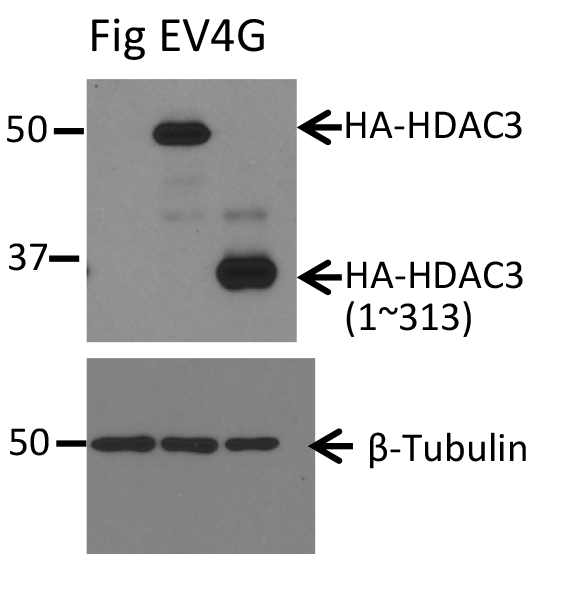

Supplement: Supplementary file 3 — Source Data for Expanded View [file EMMM-10-e8478-s011.zip › Source_data_EV4.tif]

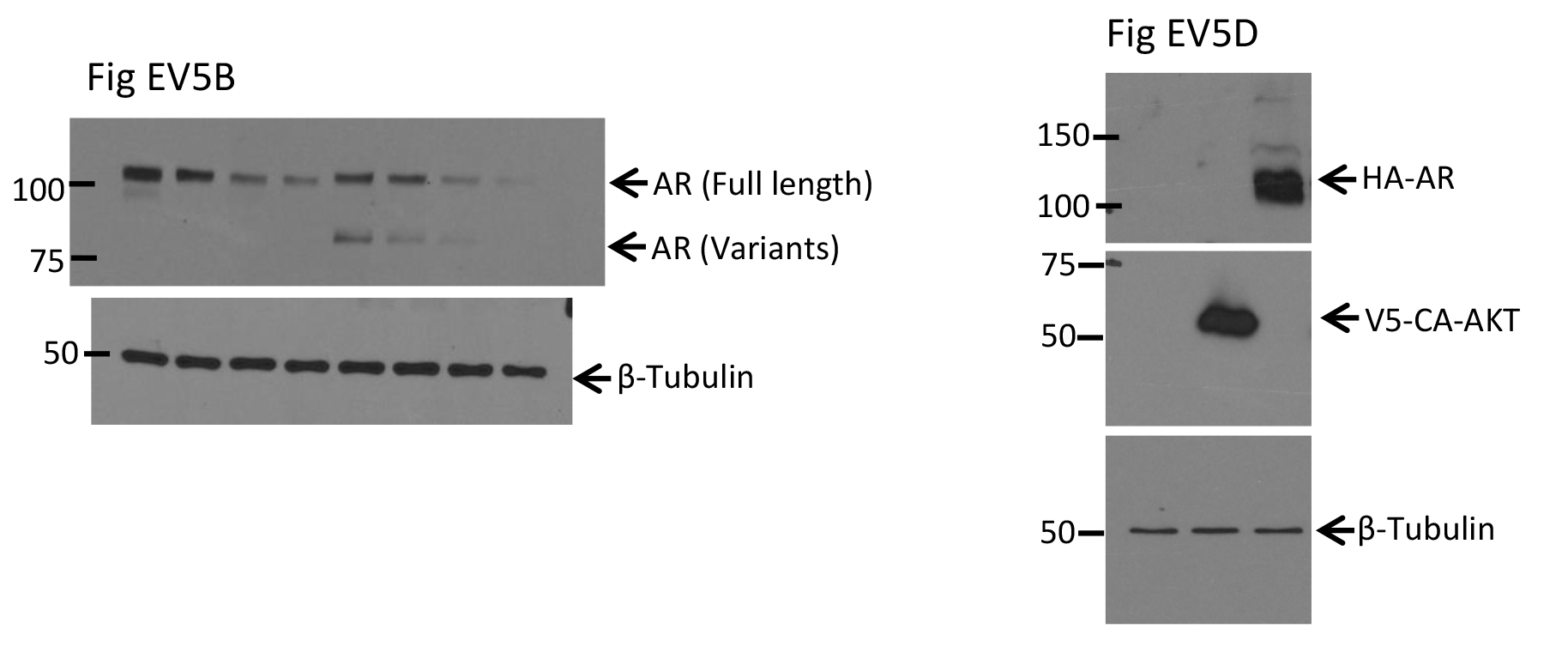

Supplement: Supplementary file 3 — Source Data for Expanded View [file EMMM-10-e8478-s011.zip › Source_data_EV5.tif]

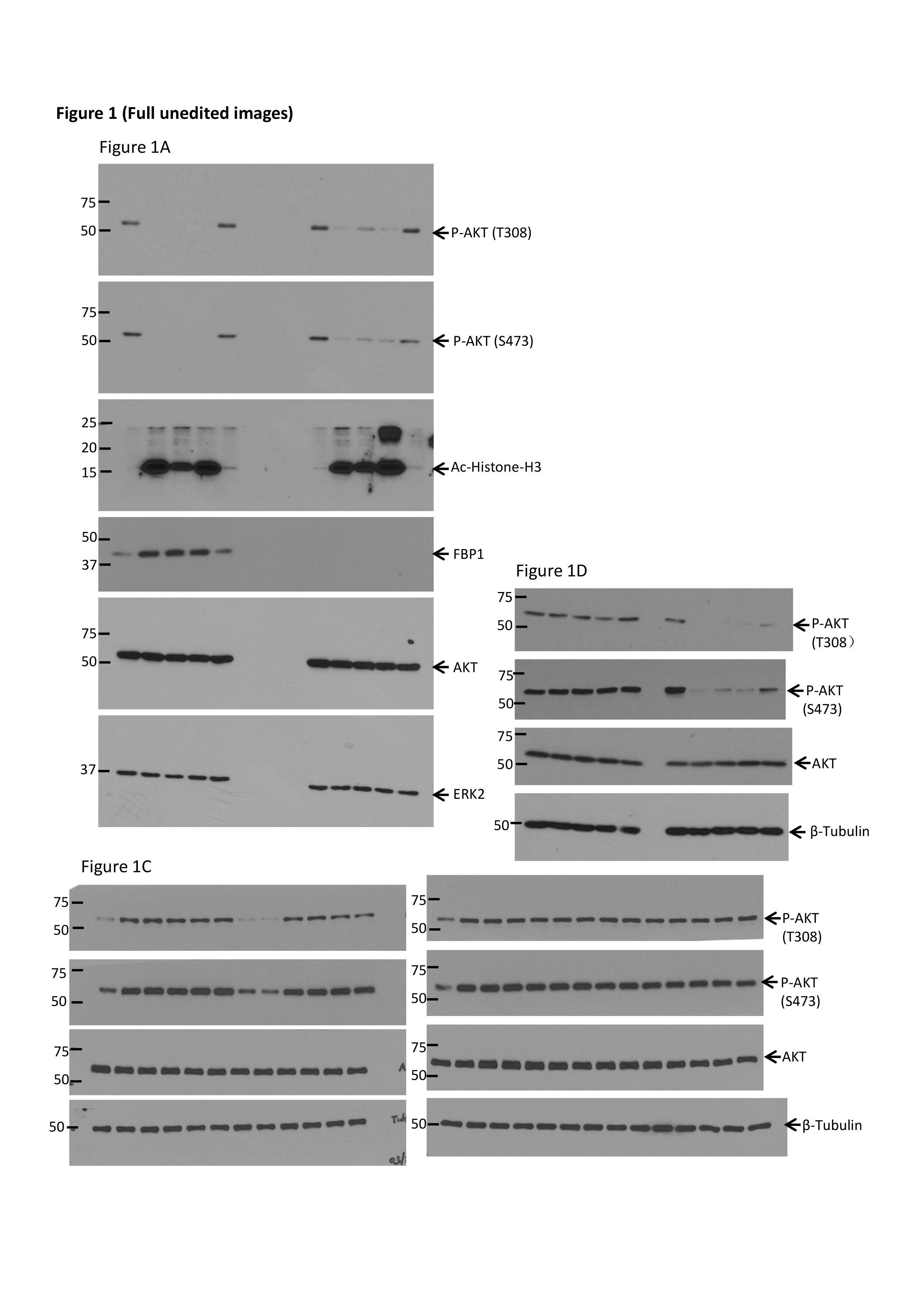

Supplement: Supplementary file 5 — Source Data for Figure 1 [file EMMM-10-e8478-s003.tif]

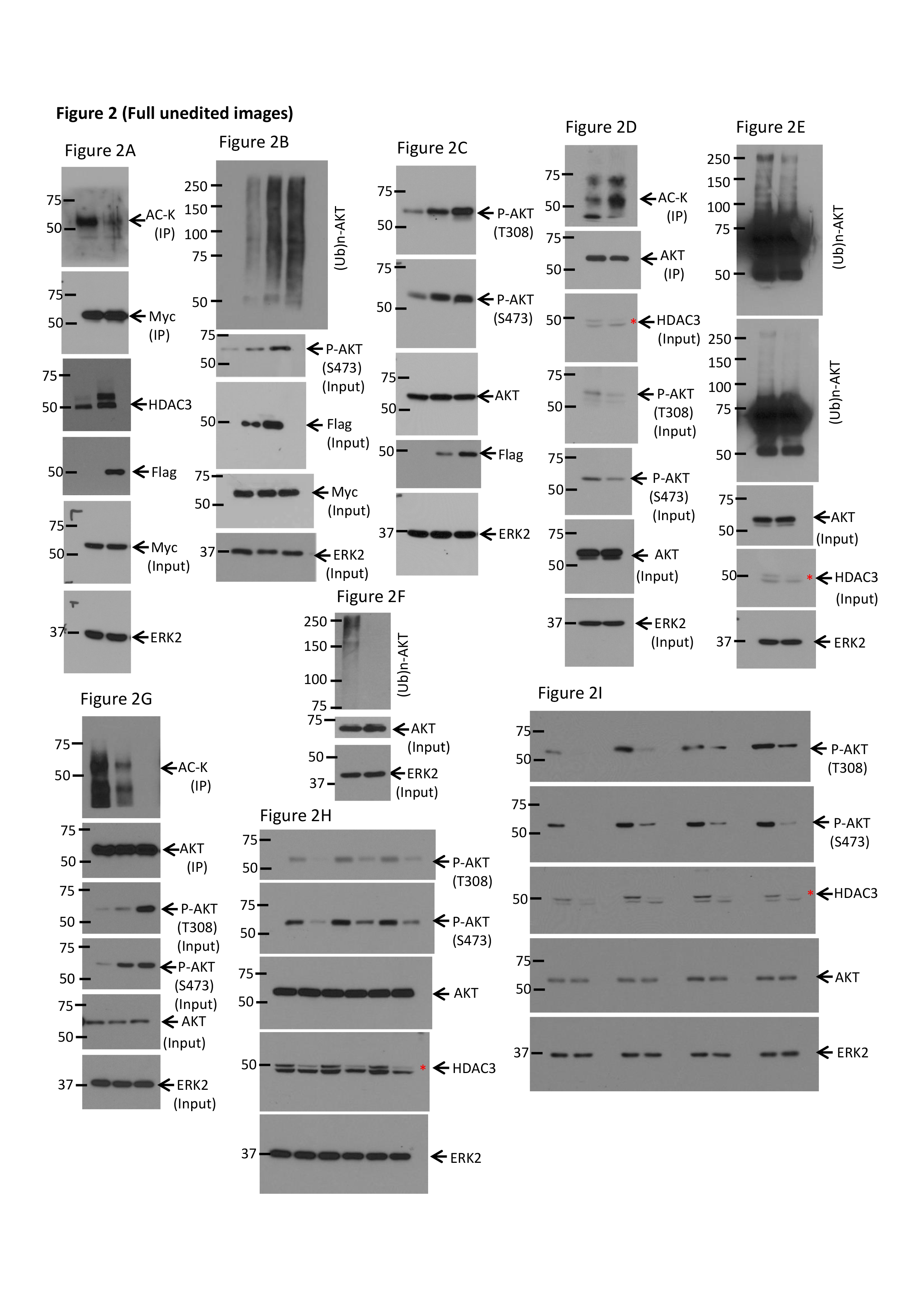

Supplement: Supplementary file 6 — Source Data for Figure 2 [file EMMM-10-e8478-s004.tif]

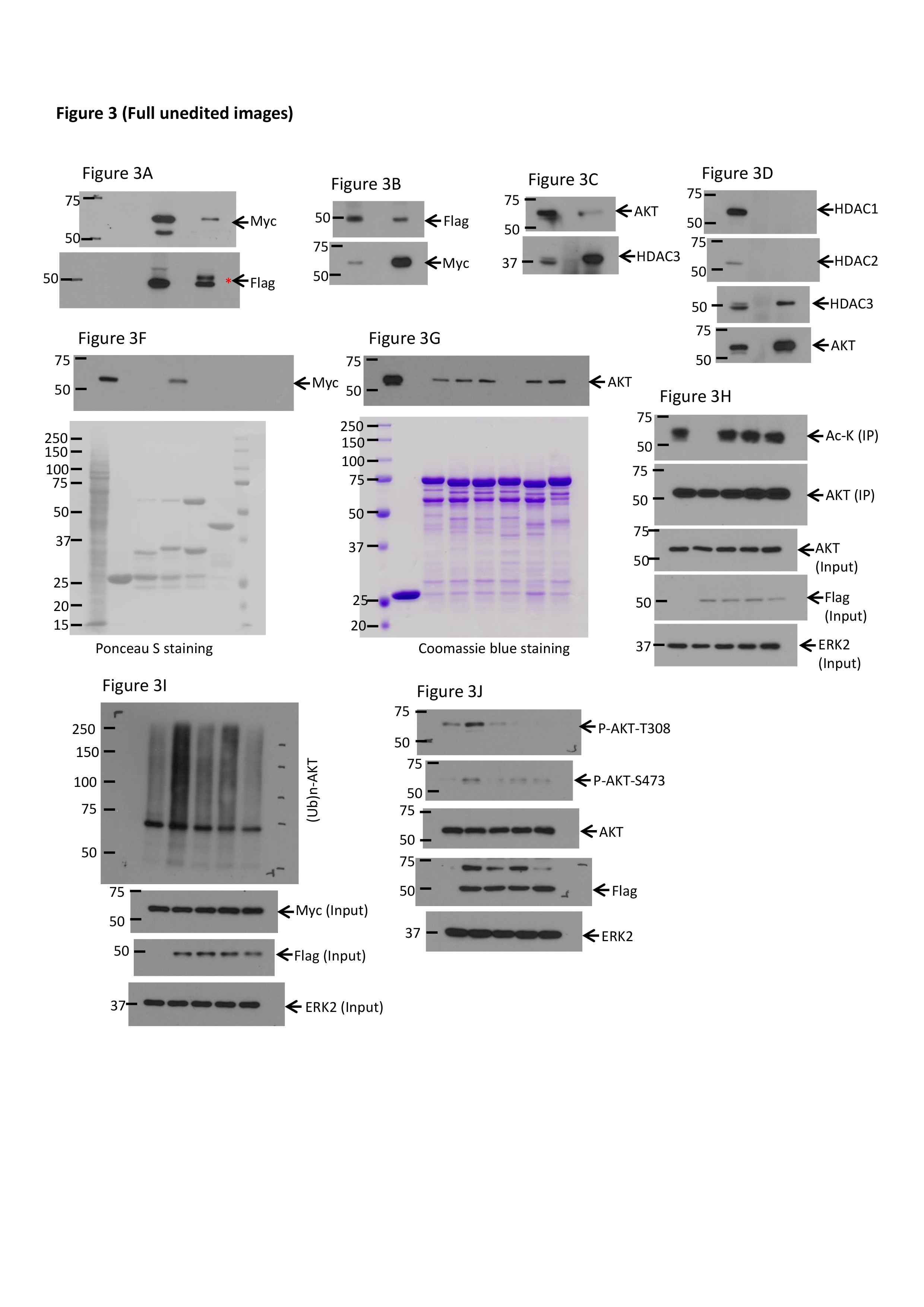

Supplement: Supplementary file 7 — Source Data for Figure 3 [file EMMM-10-e8478-s005.tif]

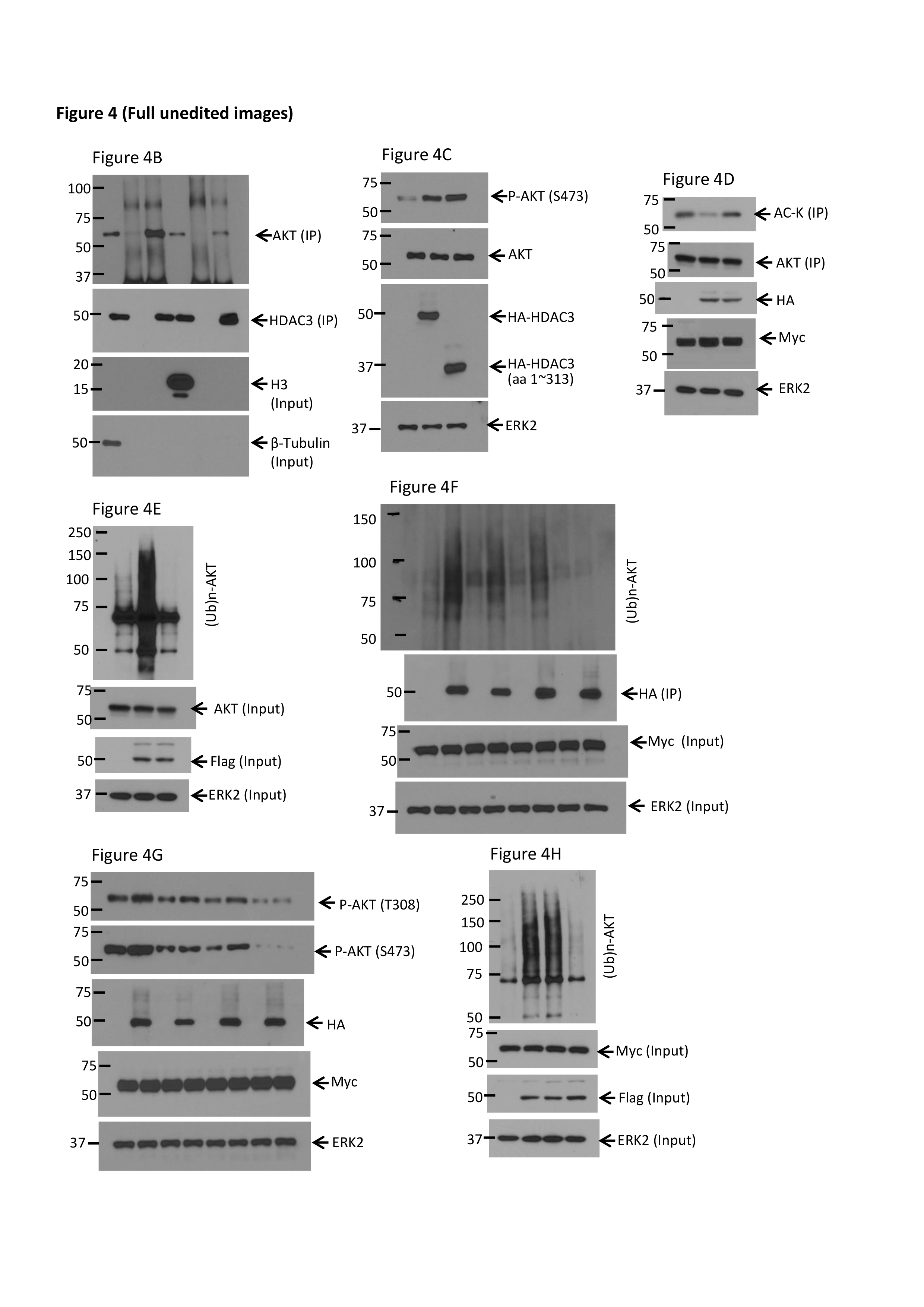

Supplement: Supplementary file 8 — Source Data for Figure 4 [file EMMM-10-e8478-s006.tif]

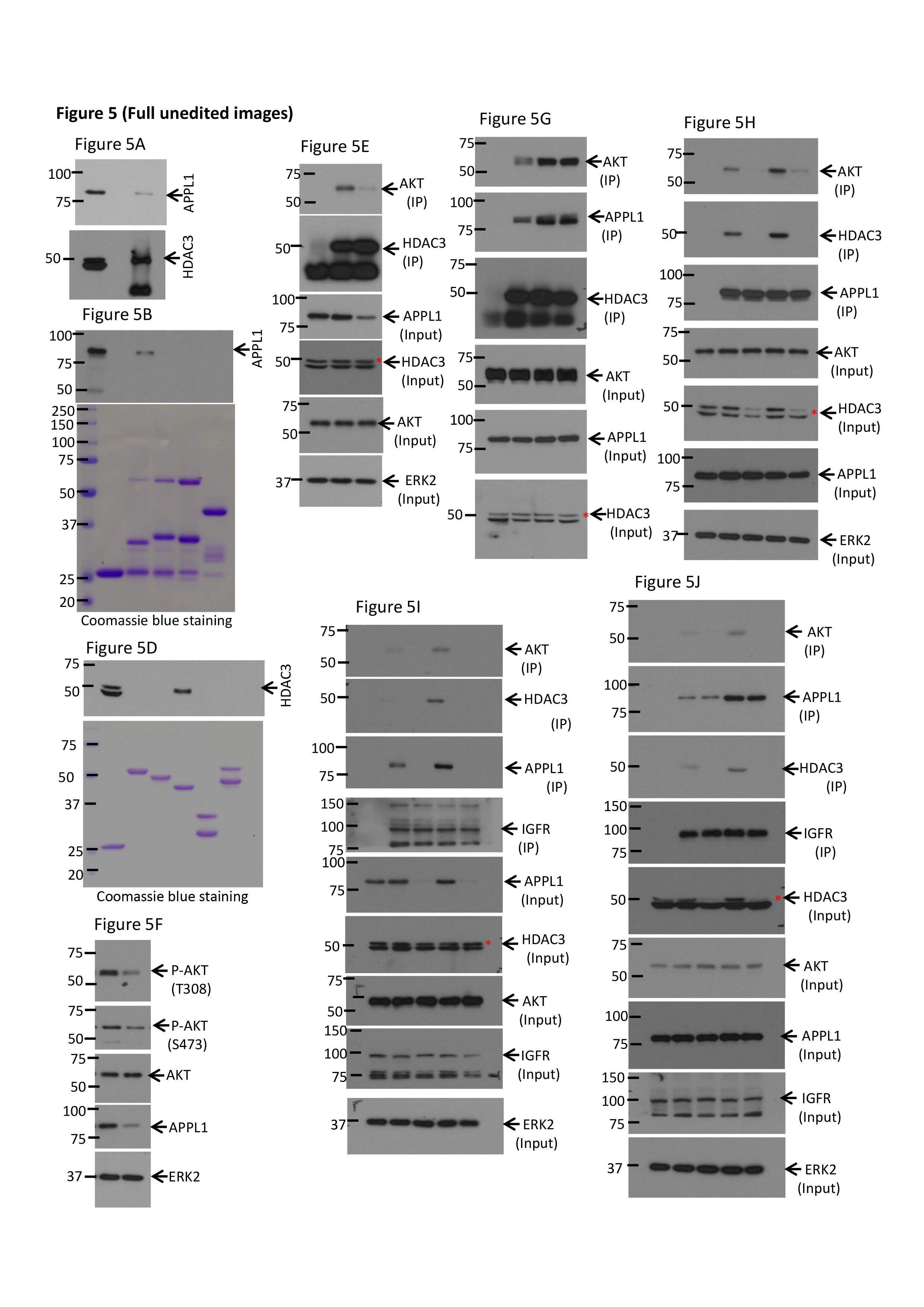

Supplement: Supplementary file 9 — Source Data for Figure 5 [file EMMM-10-e8478-s007.tif]

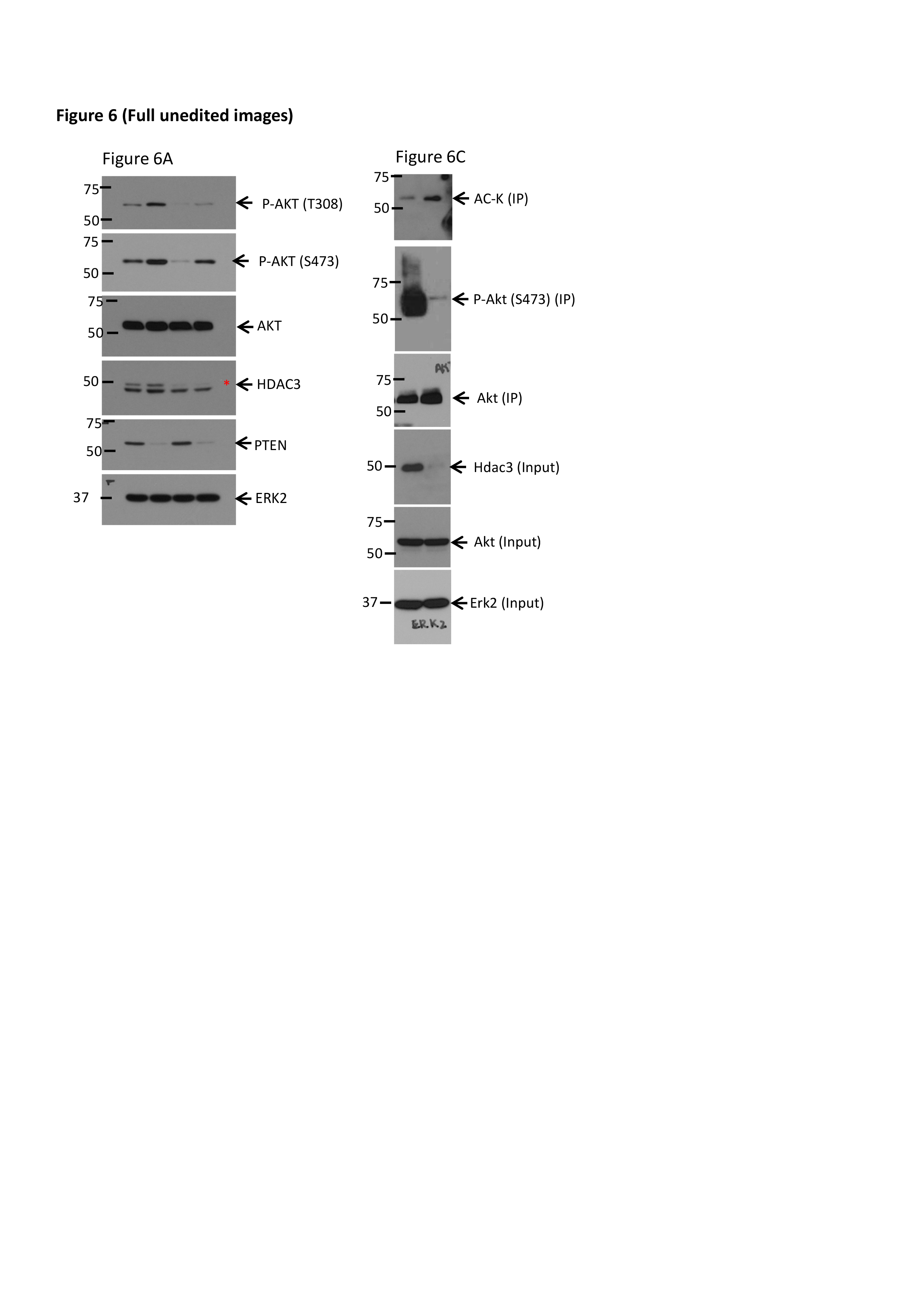

Supplement: Supplementary file 10 — Source Data for Figure 6 [file EMMM-10-e8478-s008.tif]

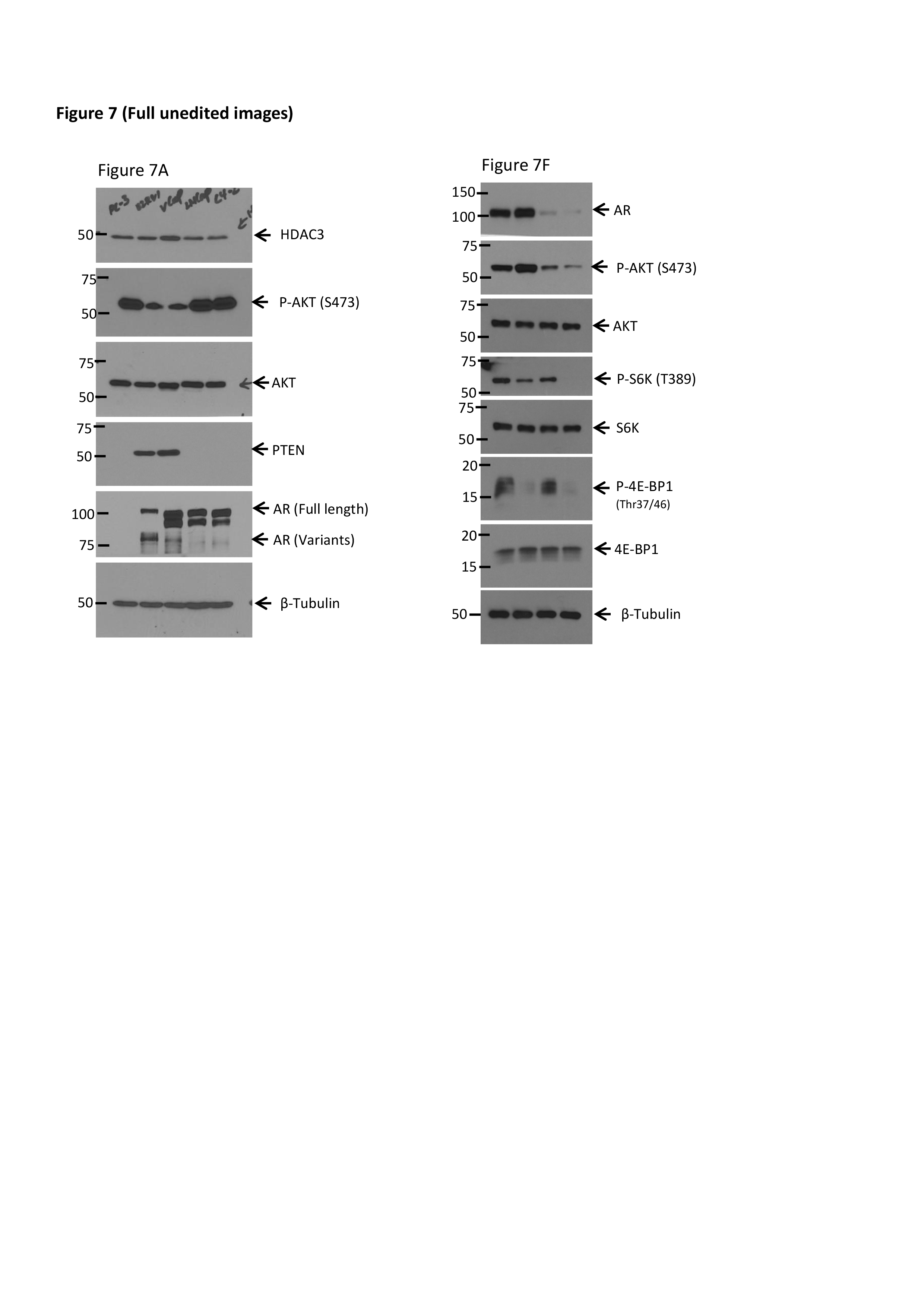

Supplement: Supplementary file 11 — Source Data for Figure 7 [file EMMM-10-e8478-s009.tif]

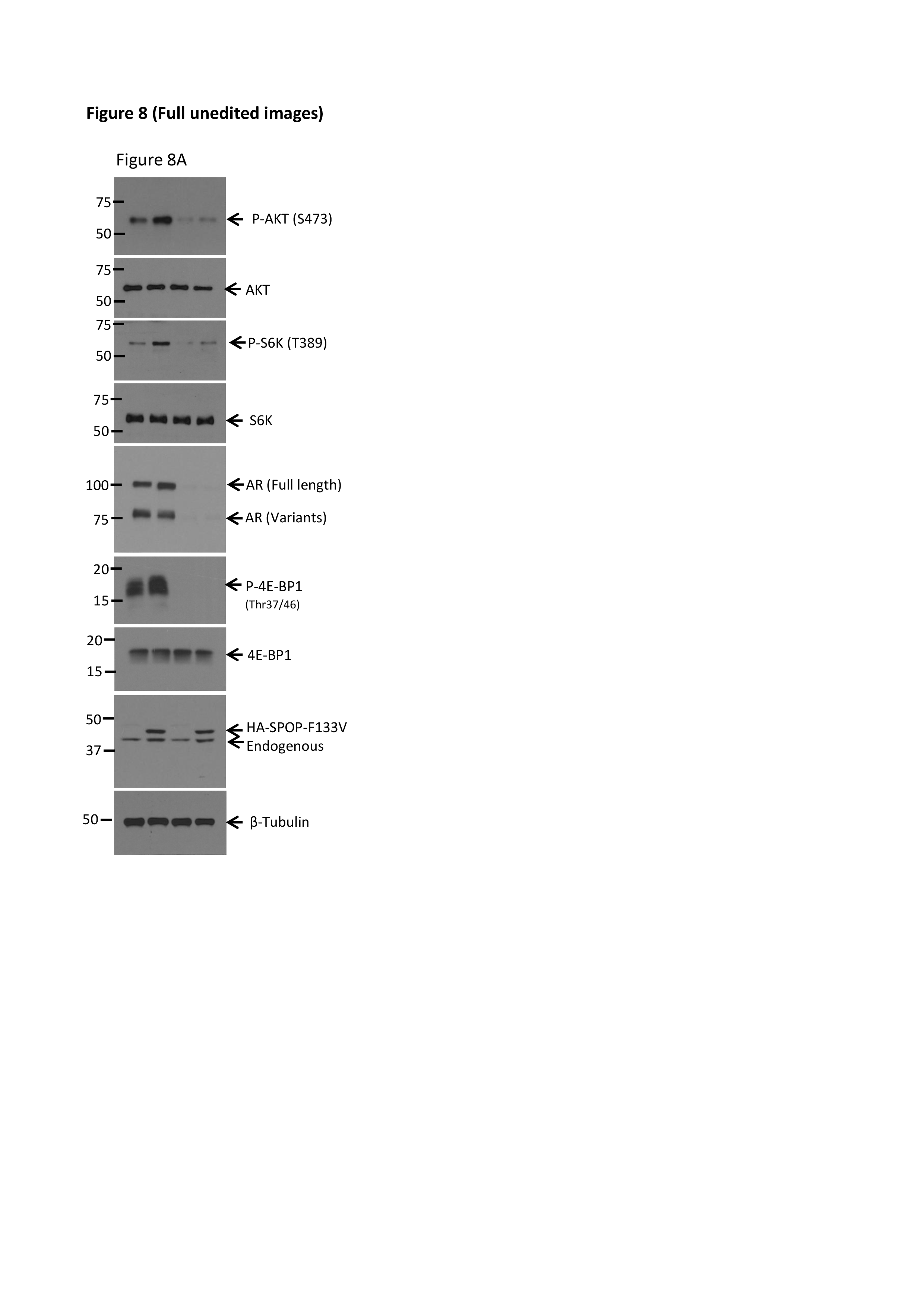

Supplement: Supplementary file 12 — Source Data for Figure 8 [file EMMM-10-e8478-s010.tif]
